# Nanostructured Hybrid BioBots for Beer Brewing

**DOI:** 10.1021/acsnano.2c12677

**Published:** 2023-04-12

**Authors:** Roberto Maria-Hormigos, Carmen C. Mayorga-Martinez, Tomáš Kinčl, Martin Pumera

**Affiliations:** † Future Energy and Innovation Laboratory, Central European Institute of Technology, Brno University of Technology (CEITEC-BUT), Purkyňova 123, Brno, 612 00 Czech Republic; ‡ Center for Advanced Functional Nanorobots, Department of Inorganic Chemistry, University of Chemistry and Technology Prague, Technická 5, Prague 6, 166 28 Czech Republic; § Department of Biotechnology, University of Chemistry and Technology Prague, Technická 5, Prague 6, 166 28 Czech Republic; ∥ Faculty of Electrical Engineering and Computer Science, VSB - Technical University of Ostrava, 17. listopadu 2172/15, Ostrava, 708 00 Czech Republic; ⊥ Department of Medical Research, China Medical University Hospital, China Medical University, No. 91 Hsueh-Shih Road, 40402 Taichung, Taiwan

**Keywords:** biohybrid, robots, magnetic, driven, beer, fermentation, hydrogel, brewing

## Abstract

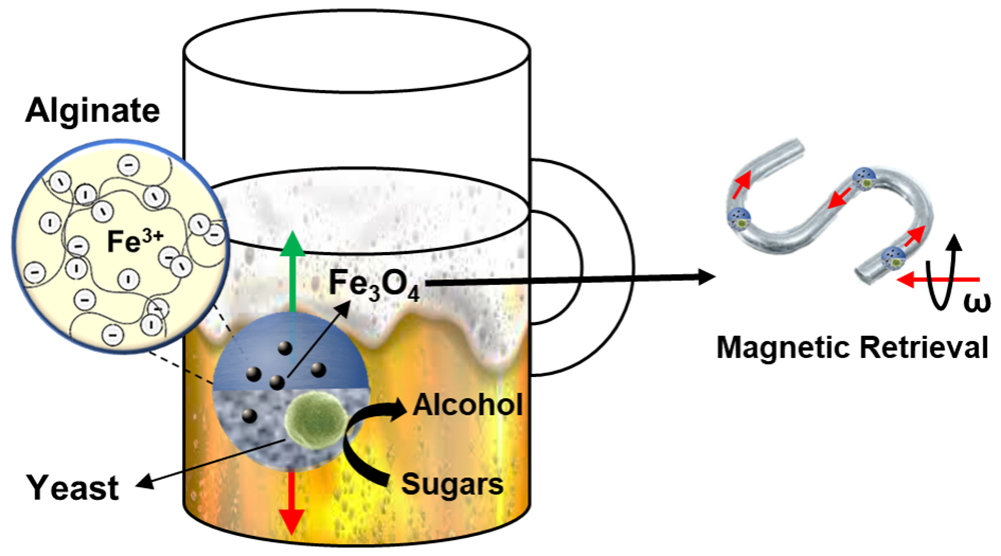

The brewing industry will amass a revenue above 500 billion euros in 2022, and the market is expected to grow annually. This industrial process is based on a slow sugar fermentation by yeast (commonly *Saccharomyces cerevisiae*). Herein, we encapsulate yeast cells into a biocompatible alginate (ALG) polymer along Fe_3_O_4_ nanoparticles to produce magneto/catalytic nanostructured ALG@yeast-Fe_3_O_4_ BioBots. Yeast encapsulated in these biocompatible BioBots keeps their biological activity (growth, reproduction, and catalytic fermentation) essential for brewing. Catalytic fermentation of sugars into CO_2_ gas caused a continuous oscillatory motion of the BioBots in the solution. This BioBot motion is employed to enhance the beer fermentation process compared to static-free yeast cells. When the process is finished, magnetic actuation of BioBots is employed for their retrieval from the beer samples, which avoids the need of additional filtration steps. All in all, we demonstrate how an industrial process such as beer production can be benefited by miniaturized autonomous magneto/catalytic BioBots.

The brewing industry will amass a revenue of more than 500 billion euro in 2022, and the market is expected to grow annually by 5.5%.^1^ Beer production is based on the sugar fermentation from malt, rice, wheat, or other sugar-rich sources into alcohol by yeast.^2^ Among the several families of yeast, *Saccharomyces cerevisiae* is the most common yeast employed for alcoholic fermentation in the brewing industry.^3^ In the case of ale beers, the fermentation takes a few days (3–4) and maturation (under pressure) 1 week. In the case of lagers the fermentation can take from 5 to 9 days, and maturation from 1 to 4 weeks.^4^ Moreover, during this process, beer can suffer from yeast spoilage, which causes economical losses.^5−7^ One way to minimize beer’s yeast spoilage is their encapsulation or immobilization to supports.^8−10^ Interestingly, cell encapsulation and immobilization on supports is one of the main strategies for biohybrid micromotors/bots synthesis.^11−15^ Micromotors/bots are active self-propelled devices that can enhance catalytic processes and reduce bio/chemical reaction times due to their propulsion and mixing enhancement.^16−18^ Furthermore, magnetic materials are commonly used in micromotors/bots design for their magnetic actuation and retrieval from the media after the process ends.^19−26^ Such magnetic micromotors/bots have been employed for pollutant degradation schemes,^20−24^ microorganism isolation from water and food,^25^ and enhancement of biocatalytic processes in food.^26^

In this work, yeast cells and Fe_3_O_4_ nanoparticles were encapsulated into alginate (ALG) particles to fabricate magneto/catalytic nanostructured ALG@yeast-Fe_3_O_4_ BioBots for the beer production process. BioBots enhanced alcoholic fermentation owing to their biocatalytic propulsion in the solution. Moreover, yeast was easily removed at the end of the fermentation process using BioBots’ magnetic actuation without additional filtration steps. BioBots were prepared by sodium-ALG chelation with iron(III) ions in the presence of yeast cells and magnetic Fe_3_O_4_ nanoparticles to confer their catalytic and magnetic propulsion, respectively. ALG is a soluble biopolymer in water obtained from algae cell walls that in the presence of divalent and trivalent cations precipitates into biocompatible capsules in which yeast can grow and keep their biological activity.^27−30^ The role of yeast is to catalyze sugars into ethanol and CO_2_ to produce beer from wort and to promote the BioBots’ catalytic vertical propulsion. The catalytic vertical propulsion of ALG@yeast-Fe_3_O_4_ BioBots was created by a buoyancy shift approach. CO_2_ bubbles, from alcoholic fermentation, were trapped in ALG@yeast-Fe_3_O_4_ BioBots and propelled toward the solution surface by buoyancy force. Then, CO_2_ bubbles were released from the BioBots, making them descend to the bottom of the flask. This process of CO_2_ entrapment–release needed a special porous Janus structure in the BioBots to obtain a constant vertical motion in the solution of the BioBots. Finally, after the alcoholic fermentation, ALG@yeast-Fe_3_O_4_ BioBots stayed in the bottom of the beer solution, and they were retrieved by BioBot magnetic actuation avoiding the filtration process to remove the yeast from the beer. Scheme 1 illustrates the whole process employed for nanostructured ALG@yeast-Fe_3_O_4_ BioBot alcoholic fermentation. First, BioBots were synthesized by ALG precipitation and yeast and Fe_3_O_4_ entrapment in an FeCl_3_ solution (A). Then, porous Janus ALG@yeast-Fe_3_O_4_ BioBots were prepared by pH gradients generated electrochemically (B) to obtain catalytic BioBots with a buoyancy shift mechanism. Afterward, BioBots were used to enhance the beer fermentation process by their catalytic vertical motion (C). Finally, BioBots were retrieved from the beer by their magnetic actuation (D) to obtain the final beer product without the necessity of yeast filtration (E).

**Scheme 1:**
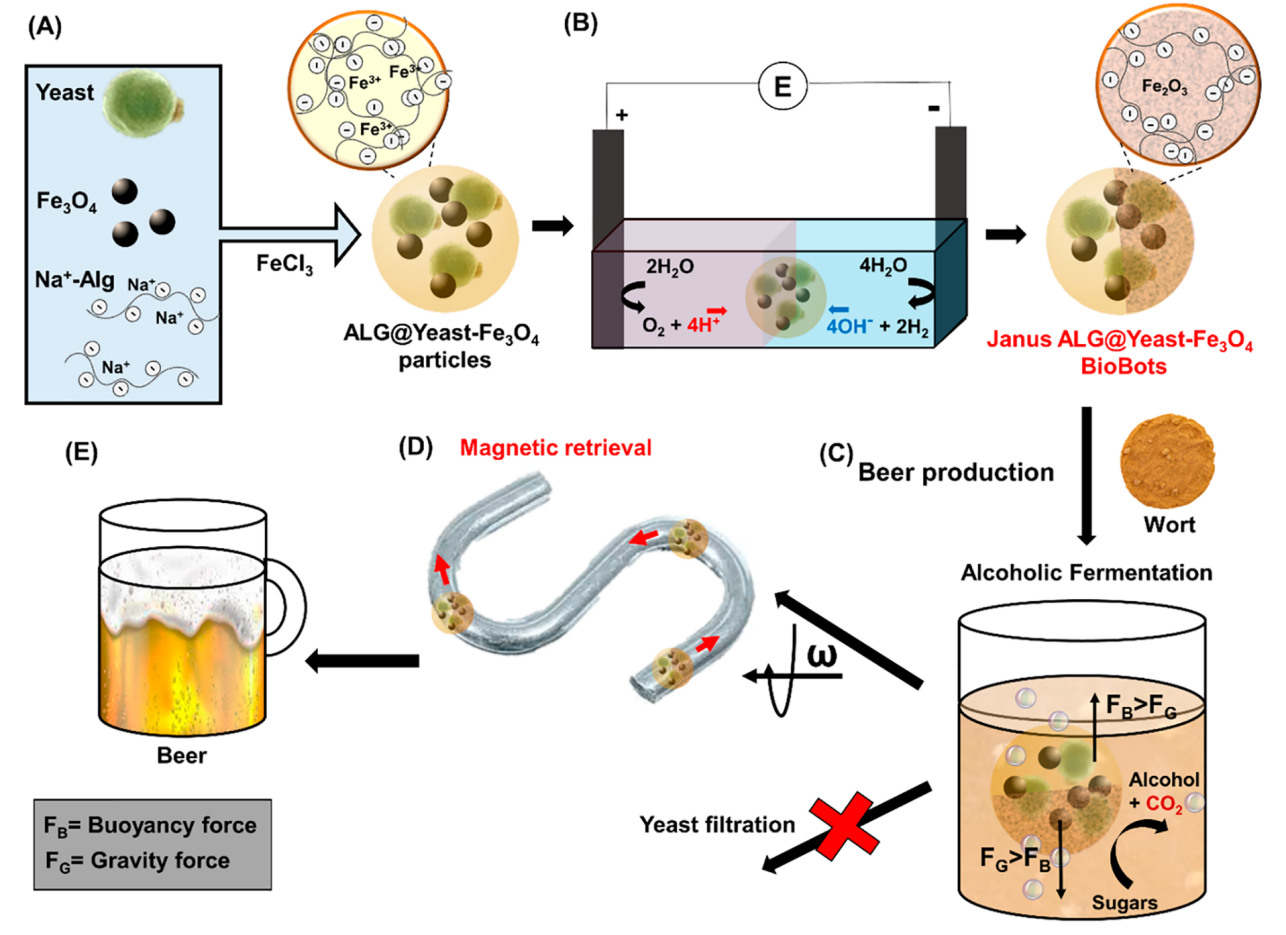
Magneto/catalytic Janus ALG@yeast-Fe_3_O_4_ BioBot development for brewing. (A) ALG@yeast-Fe_3_O_4_ BioBot synthesis by ALG precipitation in an FeCl_3_ solution and yeast and magnetic particles entrapment; (B) porous Janus structure obtained on one side of the BioBots by electrochemically generated pH gradients; (C) beer fermentation enhancement by BioBots’ catalytic vertical motion during the alcohol production process; (D) BioBot magnetic retrieval (and the yeast in them) by a rotational magnetic field; (E) final beer product obtained without filtration steps to eliminate the yeast cells.

## Results and Discussion

**Figure 1:**
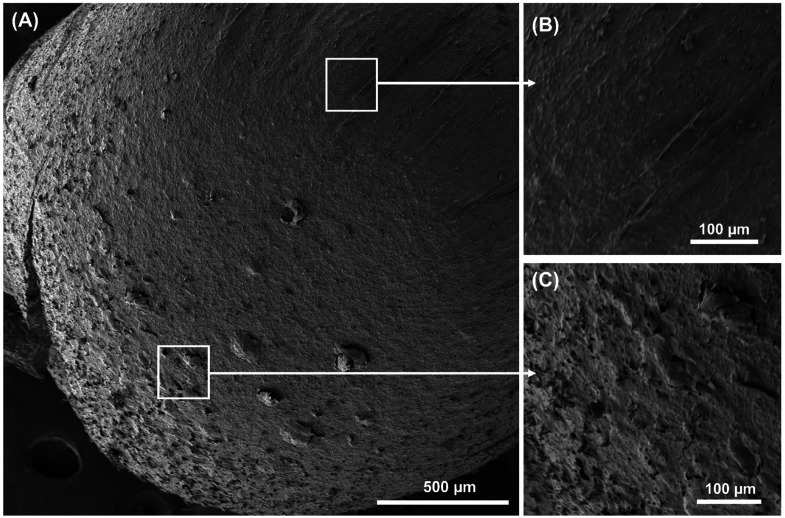
BioBot SEM characterization. (A) Janus ALG@yeast-Fe_3_O_4_ BioBot. (B) Surface magnification of the smooth hemisphere. (C) Surface magnification of the porous hemisphere.

Yeast cells and magnetic particles were encapsulated into an ALG polymeric matrix to design magneto/catalytic BioBots with a vertical motion in solution and magnetic actuation. Such BioBots were obtained by nanocomponent assembly in a precipitation method.^31^ The nanostructured BioBots were made by the assembly of Fe_3_O_4_ nanoparticles (magnetic actuation) and alginate nanochains interconnected by ion cross-linking (skeleton). Moreover, a few micrometer yeast cells were incorporated during the BioBot assembly as a functional material to produce beer. Briefly, sodium alginate, yeast cells, and Fe_3_O_4_ magnetic nanoparticles were mixed to obtain a homogeneous suspension. Then, drops of this suspension were added into an FeCl_3_ solution. In contact with Fe^3+^ cations, ALG cross-linked and immediately solidified into spheres, trapping yeast cells and Fe_3_O_4_ nanoparticles inside of them (Fe^3+^-ALG@yeast-Fe_3_O_4_ beads).^31^ Finally, a Janus microporosity structure is promoted in ALG@yeast-Fe_3_O_4_ beads to confer catalytic vertical motion in sugar solutions (ALG@yeast-Fe_3_O_4_ BioBots). The Janus porosity structure was obtained by pH gradients electrochemically generated in a two-electrode cell (Figure S1). Water electrolysis in the two-electrode cells generates protons in the anode (2H_2_O → O_2_ + 4H^+^ + 4e^–^) and hydroxyl anions in the cathode (2H_2_O + 2e^–^ → H_2_ + 2OH^–^) that migrate from the electrodes to the middle of the electrochemical cell, forming a pH gradient. When Fe^3+^-ALG@yeast-Fe_3_O_4_ beads were placed in the middle of such a pH gradient, one hemisphere of the particles got exposed to an acid media; meanwhile the other hemisphere was exposed to an alkaline media. In the alkaline media Fe^3+^ used to cross-link the ALG matrix precipitates and was removed from the ALG matrix in the form of Fe_2_O_3_. This resulted in a porosity increase in the hemisphere of the particle exposed to the alkaline media.^31^Figure 1A displays scanning electron microscopy (SEM) images of Janus ALG@yeast-Fe_3_O_4_ BioBots. Figure 1B and C show the smooth and rough hemispheres at high magnification of the ALG@yeast-Fe_3_O_4_ BioBot, respectively.

Figure S2 shows SEM images of ALG@yeast-Fe_3_O_4_ particles without the pH-gradient treatment. As can be seen, a homogeneous smooth surface along the particle was observed. Moreover, the increment in the porosity and the volume of the pores of Janus ALG@yeast-Fe_3_O_4_ BioBots was corroborated by Brunauer–Emmett–Teller (BET) characterization. Figure S3 displays BET isotherms of ALG@yeast-Fe_3_O_4_ BioBots before and after the pH treatment. Pore volume values of 0.069 and 0.207 cm^3^/g were obtained from the BET analysis of ALG@yeast-Fe_3_O_4_ BioBots before and after the pH treatment, respectively.

**Figure 2:**
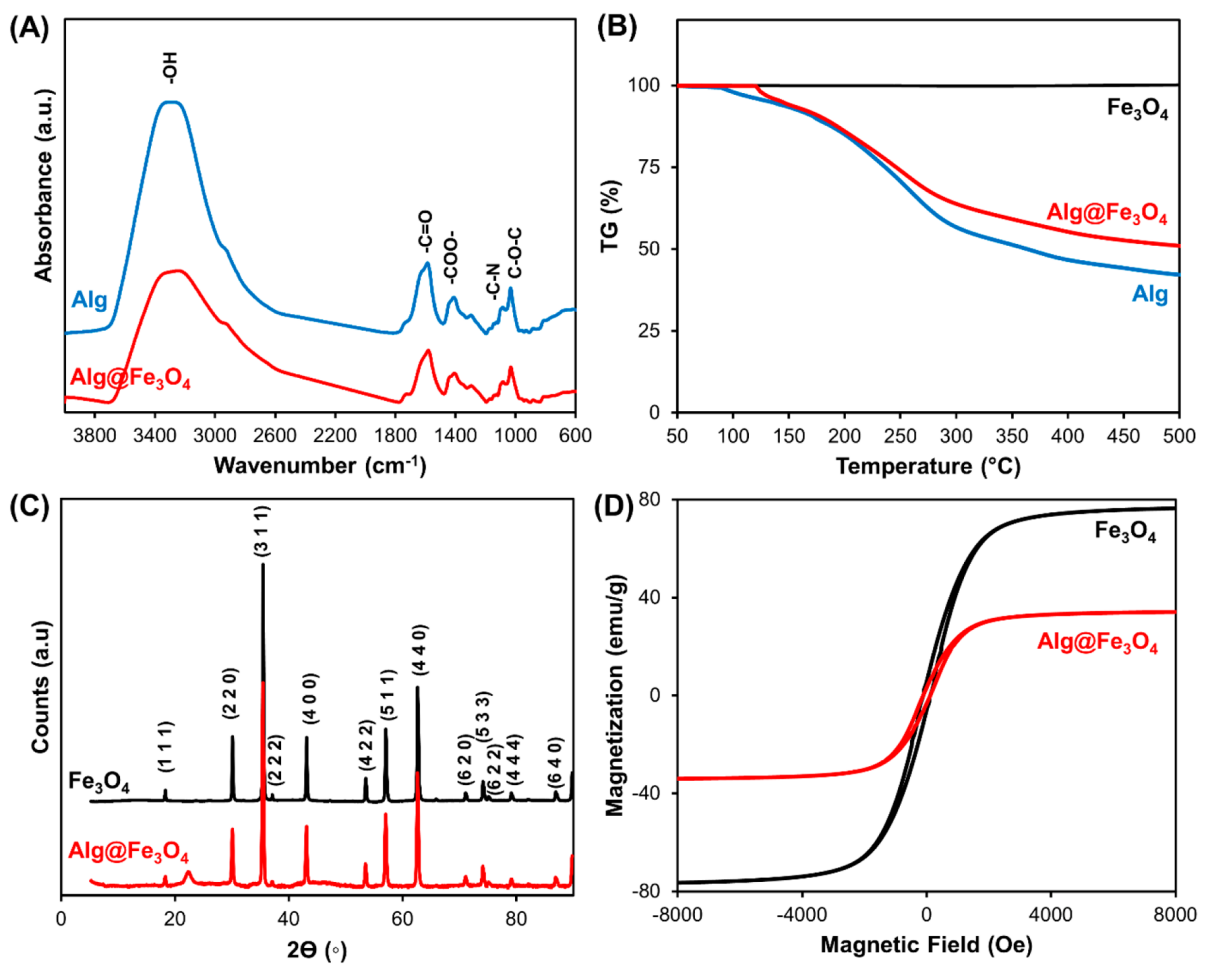
ALG@yeast-Fe_3_O_4_ BioBot characterization. (A) FTIR spectra of ALG polymer (blue line) and ALG@yeast-Fe_3_O_4_ BioBots. (B) TGA curves of Fe_3_O_4_ nanoparticles (black line), ALG beads (blue line), and ALG@yeast-Fe_3_O_4_ BioBots (red line). (C) XRD diffractograms of Fe_3_O_4_ nanoparticles (black line) and ALG@yeast-Fe_3_O_4_ BioBots (red line). (D) Magnetic hysteresis loops of ALG@yeast-Fe_3_O_4_ BioBots (red line) and Fe_3_O_4_ nanoparticles (black line).

Additional characterization of ALG@yeast-Fe_3_O_4_ BioBots was conducted to ensure that all components assembled into the spherical structures and the BioBot homogeneous production. Figure S4 displays a digital photograph of many BioBots that showed a homogeneous size with a media of 2.76 ± 0.14 mm (RSD = 5.1%, *n* = 64). In this work, BioBot size was controlled by drops of 10 μL volume with a micropipet. Figure S5 displays the whole BioBots’ morphological structure and their energy-dispersive spectrometry (EDS) mapping images. Elementary mapping of EDS analysis shows the presence of Fe element from Fe_3_O_4_ nanoparticles. Figure 2A reveals BioBot characterization by infrared spectroscopy (FTIR). FTIR was employed to determine the vibrational modes of ALG polymer on the BioBots and compare to pristine ALG. As can be seen, BioBots (red line) showed the characteristic peaks that correspond to ALG (blue line). In both spectra, a broad band (3650–3000 cm^–1^) from O–H stretching vibrations was observed. Moreover, a peak at around 2950 cm^–1^ over the O–H band from the oscillation band of −CH alkyl groups was observed as well as −C=O stretching vibrations from the carboxylic groups at 1600 cm^–1^. Asymmetric absorption bands at 1400 cm^–1^ from oscillatory COO– indicate the carboxylic groups of ALG and, owing to C–N and −C–O–C– bonds of the ALG structure, two bands in the region from 1060 to 1025 cm^–1^ were observed.^32^ Thermogravimetric analysis (TGA) of the BioBots was conducted (Figure 2B). TGA curves of Fe_3_O_4_ nanoparticles showed thermal stability without any mass loss at all observed temperatures (black line). The main loss of mass of ALG@yeast-Fe_3_O_4_ BioBots (red line) and the ALG beads (blue line) was at 180 and 300 °C (49% and 57%, respectively) owing to the ALG chains’ thermal decomposition.^33^

Crystallinity of Fe_3_O_4_ magnetic nanoparticles from the BioBots was characterized by X-ray diffraction (XRD) (Figure 2C). As can be seen, BioBots (red line) displayed a magnetite crystalline structure of Fe_3_O_4_ (black line) with peaks at 2θ values of 18.31°, 30.11°, 35.47°, 37.05°, 43.13°, 53.49°, 57.34°, 62.61°, 71.06°, 74.09°, 75.17°, 79.08°, and 86.89° (ref code 01-071-6336).^34^ BioBots’ magnetic properties were characterized by a sample vibrating magnetometer at 300 K (Figure 2D). BioBots and Fe_3_O_4_ nanoparticles showed a magnetic hysteresis loop with a small coercivity of 112 Oe, suggesting a soft ferromagnetic behavior. The saturation magnetization value (*M*_s_) of the ALG@yeast-Fe_3_O_4_ BioBots was 34.1 emu/g compared to 76.5 emu/g of pristine Fe_3_O_4_ nanoparticles owing to the decrease of Fe_3_O_4_ nanoparticle concentration on the BioBots (40% w/w).^34^ These magnetic properties allow BioBot magnetization independently of the alginate matrix and their subsequent magnetic actuation under a rotational magnetic field.

**Figure 3:**
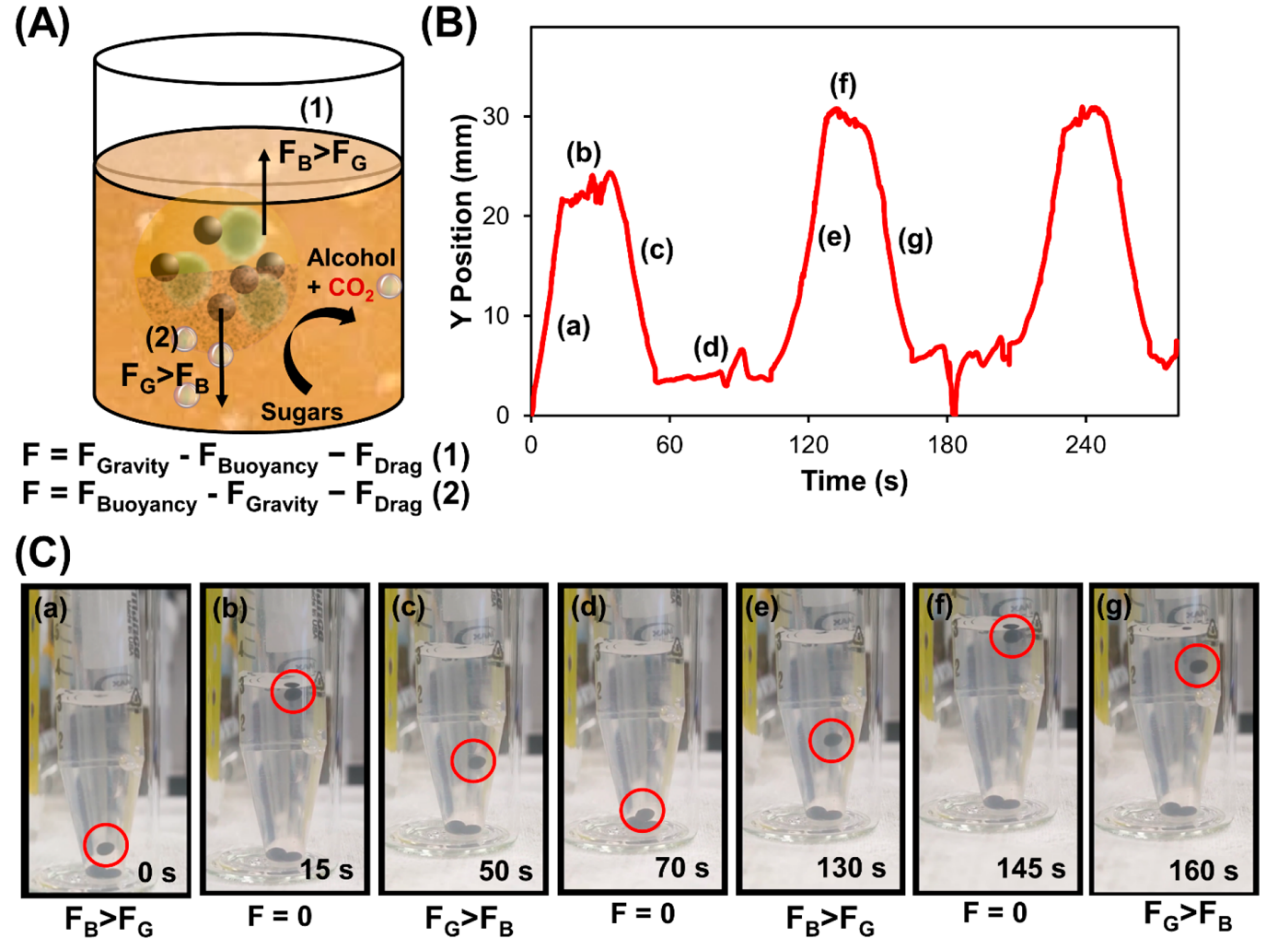
(A) Scheme of Janus ALG@yeast-Fe_3_O_4_ BioBot propulsion by a buoyancy shift mechanism. (B) Position of the Janus ALG@yeast-Fe_3_O_4_ BioBot over time from Video S1 at real-time speed. (C) Time-lapse images extracted from Video S1. Lowercase letters (a–g) correlate the particles’ position with their forces in each moment.

Yeast incorporation into the BioBots structure was confirmed by the catalytic propulsion and beer fermentation of BioBots. ALG@yeast-Fe_3_O_4_ BioBots followed a catalytic buoyancy shift mechanism for their vertical motion in sugar solutions. The buoyancy shift mechanism is based on the periodic entrapment and release of bubbles from a particle to cause a vertical motion in solution.^35^ The forces involving this mechanism reported by Inagi’s group can be found in the Supporting Information.^35^ In our case, yeast encapsulated in the ALG matrix produces CO_2_ entrapment in the BioBots, which causes a directional vertical motion. Then, the CO_2_ was released in contact with the water–air interface, and BioBots descended in the solution by gravity. This periodic gas entrapment and release caused a constant shift in the particle’s overall buoyancy. Figure 3 and Video S1 show the directional vertical and descending motion of Janus ALG@yeast-Fe_3_O_4_ BioBots in a 3% sugar solution. When BioBots stayed at the bottom of the sugar solution, the gravity force (*F*_G_) was higher than the buoyancy force (*F*_B_) and no movement was observed. Then, as a result of CO_2_ production and entrapment inside of the BioBots, *F*_B_ increased until it was higher than *F*_G_. In this moment, the BioBots started to ascend in the solution (a, *F*_B_ > *F*_G_) until they reached the liquid–air interface (b, *F* = 0). Next, the bubbles were released, and due to simple gravity, the BioBots descended (c, *F*_G_ > F_B_) to the solution bottom (d, *F* = 0), where the motion cycle restarted (e–g). In this sense Janus ALG@yeast-Fe_3_O_4_ BioBots followed the behavior already reported in previous works using this buoyancy shift mechanism.^31,35^

Also, as in these previously works,^31,35^ Janus morphology played an essential role in BioBot propulsion, as can be seen in Figure S6 and Video S2. Non-Janus ALG@yeast-Fe_3_O_4_ particles (without pH gradient treatment) float in the solution as a consequence of the difficulty of CO_2_ release after entrapment on the ALG matrix. Particles that are too porous (treated for 30 min in the pH gradient) remained in the solution bottom due to constant release of CO_2_ from the ALG matrix, which prevented their ascension in the solution. Meanwhile, Janus BioBots (treated for the optimal 10 min in the pH gradient) can ascend in the solution by CO_2_ entrapment in the ALG matrix and then descend after CO_2_ release on the liquid–air interface owing to their controlled porosity.

**Figure 4:**
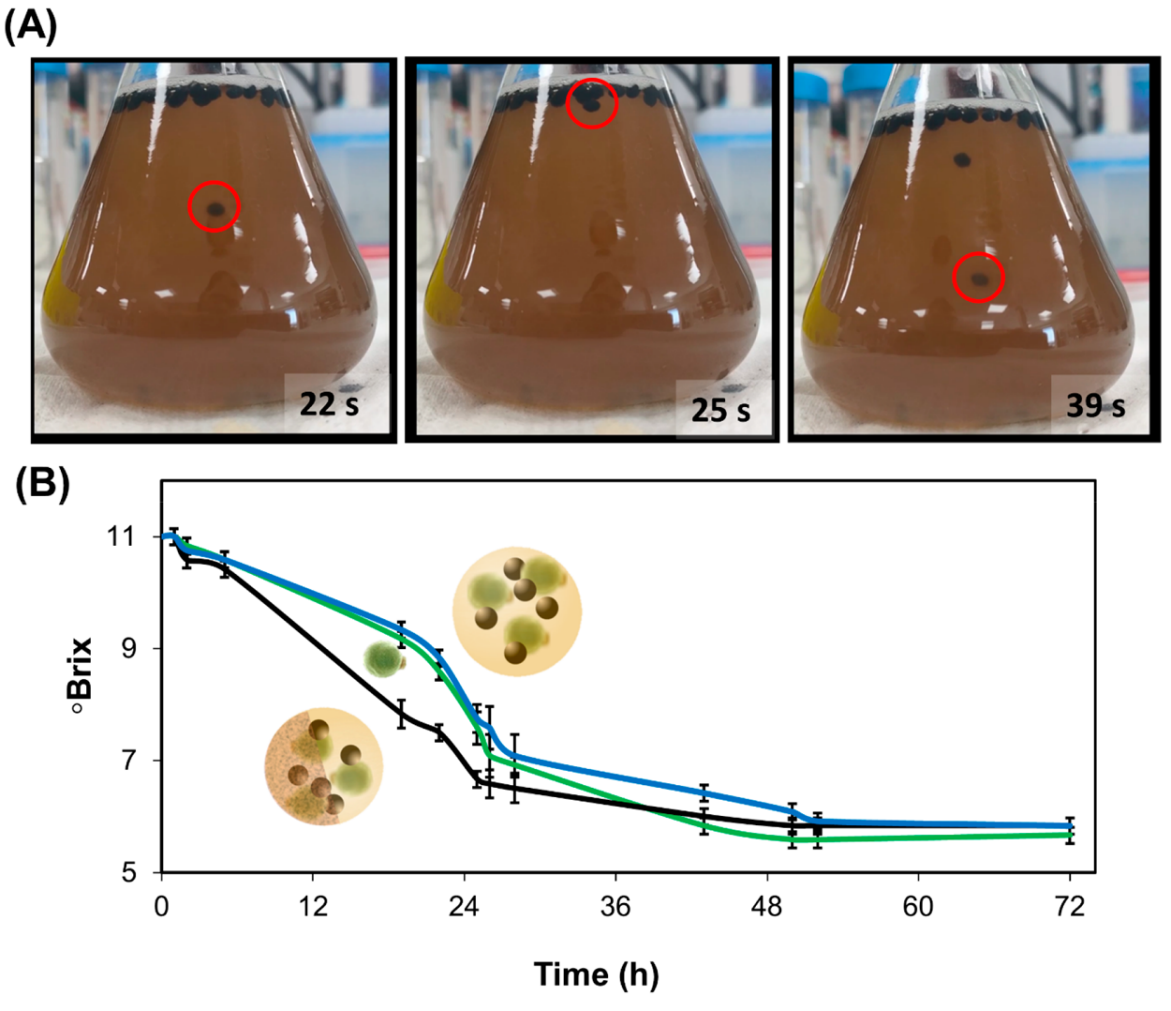
Alcoholic fermentation process. (A) Time lapse image of Janus ALG@yeast-Fe_3_O_4_ BioBots’ oscillatory motion in the wort during the fermentation process. (B) Sugar concentration monitoring as °Brix during the fermentation process using Janus ALG@yeast-Fe_3_O_4_ BioBots (black line), non-Janus floating BioBots (blue line), and free yeast (green line). Initial wort 11°Brix and inoculation concentration was 0.5 g/L of dry yeast or BioBots in all experiments, *n* = 3.

During the beer fermentation process sugar concentration can decrease from an initial value above 10% to less than 1%.^36^Video S3 shows the directional vertical motion of Janus ALG@yeast-Fe_3_O_4_ BioBots at different sugar concentrations ranging from 1% to 10%. As can be seen, oscillatory motion was observed at sugar concentrations above 3%. However, Janus ALG@yeast-Fe_3_O_4_ BioBots remain in the bottom of the solution at 1% sugar concentration. So, it was expected that BioBots can propel themselves during the whole beer fermentation process. Figure 4A and Video S4 show the oscillatory motion of Janus ALG@yeast-Fe_3_O_4_ BioBots in the wort during the beer fermentation process at 25 °C. Wort is a complex nutrient mixture extracted from barley malt. It contains fermentable sugars, especially maltose, amino acids and proteins, minerals, vitamins, and other compounds. As result of the fermentation process, sugars are consumed by yeast to produce alcohol and other flavor/aromatic compounds such as esters.^36^ Alcoholic fermentation was monitored with an analog refractometer by sugar concentration changes as a function of °Brix (one degree Brix is 1 g of sucrose in 100 g of solution).^37^Figure 4B displays °Brix decrease over time using Janus ALG@yeast-Fe_3_O_4_ BioBots, free yeast, and non-Janus ALG@yeast-Fe_3_O_4_ BioBots during a fermentation process of an initial wort 11°Brix. As can be seen, Janus ALG@yeast-Fe_3_O_4_ BioBots transform sugars into alcohol faster than free yeast and floating non-Janus BioBots due to the enhanced intermixing of their oscillatory vertical motion. Moreover, CO_2_ evolution during the fermentation process was monitored by collecting the gas produced in a graduated tube filled previously with water. Figure S7 shows the volume of CO_2_ recorded during the reaction. A higher CO_2_ volume production indicated a higher biological activity during such time. As can be seen, the increase of gas evolution correlated to the °Brix decrease in Figure 4B. This indicated that the fermentation process was taking place and showed the enhancement of the fermentation process after several hours using the Janus ALG@yeast-Fe_3_O_4_ BioBots compared to the standard free yeast process.

Normally, at the end of the process, the same final value of °Brix reflects a comparable degree of fermentation. Final °Brix obtained using moving BioBots, floating BioBots, and free yeast was compared to ensure that there were not significant differences at the end of the process. One-way analysis of variance (ANOVA) was carried out to compare the results obtained at the end of the fermentation process (72 h). An *F* value of 2.33 was obtained for the experimental means comparison, this value is smaller than the *F*_critical_ of 5.14 (2 and 6 degrees of freedom) with a significance of 0.05 (95% of confidence). This result showed that there were not statistical differences in the final value of °Brix at the end of the fermentation process between using free yeast and Janus and non-Janus BioBots.

In an ale beer fermentation process, yeast remains floating on the wort and flocculates on the bottom of the solution at the end.^36^ Flocculation was also observed in ALG@yeast-Fe_3_O_4_ BioBots after around 48 h when BioBots stopped their catalytic motion. Then, ALG@yeast-Fe_3_O_4_ BioBots can be maneuvered by magnetic actuation under a rotational magnetic field to separate them (and the yeast in them) from the beer. This allowed the separation of the yeast from the beer without additional filtration steps.

**Figure 5:**
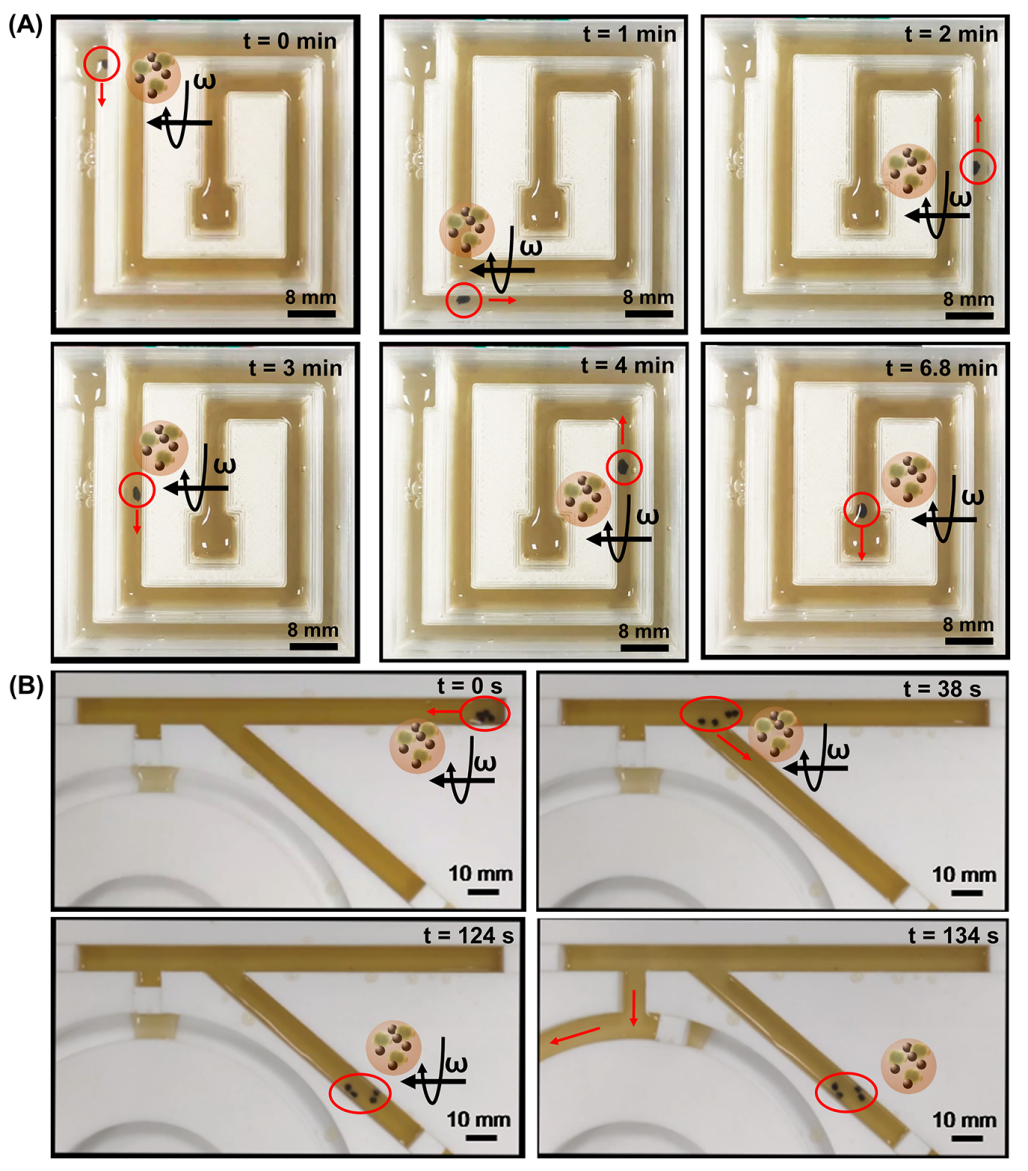
BioBots’ magnetic actuation in beer. (A) Time lapse images of ALG@yeast-Fe_3_O_4_ magnetic navigation on a complex channel. (B) Time lapse images of BioBot magnetic retrieval from a beer sample. Rotational magnetic field intensity = 40 mT.

Video S5 shows the magnetic actuation of a swarm of BioBots at different frequencies of a rotational magnetic field. It was observed that their speed of displacement was inversely proportional to the rotational frequency of the magnetic field (see Figure S8). Figure 5A and Video S6 show the magnetic navigation of an ALG@yeast-Fe_3_O_4_ BioBot in a beer solution inside of a 3D-printed channel system using a 3 Hz rotational magnetic field. It was possible to control the magnetic motion of one BioBot as well as when they were in a swarm along the channel. Figure 5B and Video S7 show BioBot magnetic retrieval from the beer sample and their magnetic swarming behavior. First, BioBots were isolated from the beer stream by their magnetic actuation to a separate channel, and then, beer was collected by opening the “tap” on the main beer stream (white piece blocking the flow of beer). In this sense, yeast was separated from the beer at the end of the fermentation process without any filtration process. Moreover, after BioBot separation, they were washed five times with water to eliminate the excess of yeast produced, and they were reused in the beer fermentation of fresh wort. As can be seen in Figure S9, BioBots’ (yeast) biocatalytic activity remains up to four beer fermentation cycles.

## Conclusions

Magneto-catalytic Janus ALG@yeast-Fe_3_O_4_ BioBots were obtained by a simultaneous ALG precipitation in an Fe(III) ion solution, yeast cells, and Fe_3_O_4_ nanoparticles. These BioBots exhibited a catalytically controlled vertical motion due to a buoyancy shift mechanism. Janus character needed for the vertical motion was obtained by using a pH gradient electrogenerated in two-electrode cells, where Fe(III) ions from ALG polymer cross-linking reacted in basic media, and they were removed from the ALG matrix network, causing porosity in the matrix in one hemisphere. Janus ALG@yeast-Fe_3_O_4_ BioBots self-propelled in sugar solutions of 3% or above. In addition, BioBots were propelled in wort solution during the whole beer fermentation process due to its sugar content. Catalytic BioBots enhanced the alcohol production compared to their static counterparts, floating BioBots, and free yeast cells. At the end of the beer fermentation process, BioBots sedimented in the solution, allowing their separation by magnetic actuation under a rotational magnetic field. The magnetic retrieval of BioBots allows the obtention of clear beer without filtration steps. The main limitation of the work is the obtention of a Janus structure on one side of the BioBots. However, the electrochemical cell employed in this work is a straightforward 3D printing design that can be scalable to industrial purposes to minimize the impact of this limitation. All in all, we have demonstrated how beer fermentation can be benefited by small autonomous BioBots.

## Methods

### Chemicals and Materials

Sodium alginate (Sigma-Aldrich, Cat. W201502), FeCl_3_ (Sigma-Aldrich, Cat. 236489), Fe_3_O_4_ nanoparticles (Sigma-Aldrich, Cat. 637106), glucose (Sigma-Aldrich, Cat. G8270), and potassium nitrate (Penta, Cat. 12970) were employed as received. Dry yeast *Saccharomyces cerevisiae* (Fermentis, SafAle S-04) and dried wort (Agra group, a.s.) were used. All solutions were prepared in tap water if not otherwise indicated.

### ALG@yeast-Fe_3_O_4_ Particle Synthesis

Sodium-ALG (2.5% w/v) was dissolved in ultrapure water. Then, 250 mg of dry yeast and 250 mg of Fe_3_O_4_ nanoparticles were mixed under mechanical stirring with 5 mL of ALG solution. The suspension was stored at 4 °C until used or yeast spoilage after 2 weeks. Particle synthesis was carried by adding 10 μL of the suspension of yeast-Fe_3_O_4_ in alginate dropwise with a micropipet in a 0.1 M FeCl_3_ solution. ALG instantly precipitated in contact with Fe(III) ions and trapped Fe_3_O_4_ nanoparticles and yeast cells due to a chelation reaction that increased ALG chain interactions. ALG@yeast-Fe_3_O_4_ particles were kept in the FeCl_3_ for 15 min to complete ALG cross-linking with the iron ions. Then, particles were cleaned three times with ultrapure water to eliminate excess Fe(III). ALG@yeast-Fe_3_O_4_ particles’ porosity was immediately modified after synthesis.

### Janus ALG@yeast-Fe_3_O_4_ BioBot Preparation

Porous Janus structures on ALG@yeast-Fe_3_O_4_ particles were obtained by an electrochemical method reported before.^31^ ALG@yeast-Fe_3_O_4_ particles were placed in a 4 cm length rectangular electrochemical cell with two platinum square planar electrodes (1.5 × 1.5 cm) attached to the extremes. Figure S1 shows a scheme of the experimental setup for more details. The electrochemical cell was filled with a 5 mM potassium nitrate solution, and water splitting was carried out in the platinum electrodes by applying a 5 V potential. As a result of water splitting, protons and hydroxyl ions were obtained in each electrode. These ions migrate by diffusion to the center of the cell, creating two pH zones in the cell. As the BioBots were in the middle of the cell, one hemisphere of the particles was exposed to the basic media and the other remained in the acid one. Iron(III) ions that cross-linked the ALG polymer were retrieved from the ALG matrix by forming insoluble Fe_2_O_3_ particles in the basic media. In consecutive reactions, Fe(III) reacts with OH^–^ anions to form Fe(OH)_*x*_ followed by fast dehydration to finally form stable Fe_2_O_3_ particles.^38^ As a consequence of this reaction, pores were opened on ALG@yeast-Fe_3_O_4_ particles and a porous Janus structure was obtained. Janus ALG@yeast-Fe_3_O_4_ BioBots were stored at 4 °C in ultrapure water until use.

### BioBot Characterization

The structural characterization of materials with high water content, such as hydrogels, is challenging due to the inherent properties of water that are dependent on conditions of temperature and pressure.^39^ ALG@yeast-Fe_3_O_4_ BioBots were freeze-dried to characterize them in their original morphology and structure. Freeze-drying was carried out by L4-110 Gregor instruments at a −90 °C temperature and a <6 mPa pressure to BioBots prefrozen in liquid nitrogen. SEM images and EDS mapping were obtained by a Tescan MAIA3 microscope equipped with an Oxford Instruments EDS detector. An acceleration voltage of 5 kV was employed to obtain the images. SEM/EDS samples were gold sputtered to make them conductive before analysis. Nitrogen absorption desorption BET surface analysis of materials was measured by a NOVAtouch Quantachrome instrument. FTIR samples were analyzed on a Nicolet 6700 FTIR spectrometer (Thermo-Nicolet, USA) in conjunction with a GladiATR diamond ATR attachment (PIKE, USA). TGA samples were analyzed on a thermobalance (Stanton Redcroft, TG-750, England) using a N_2_ atmosphere with a temperature slope of 10 °C min^–1^. XRD samples were analyzed on an X-ray diffractometer (Bruker, AXS D8, Germany) using Co as anode source. Data were transformed into a Cu reference as an anode source for representation. Magnetic characterization of the sample was performed in a VSM (Versalab, Quantum Designs, USA) at room temperature (300 K), and hysteresis loops were recorded between 0.8 and −0.8 T. BioBot size distribution was calculated by image analysis in NIS-Elements software after image calibration.

### Catalytic and Beer Fermentation Experiments

BioBot catalytic oscillatory motion in tap water solutions was achieved using different glucose concentrations. ALG@yeast-Fe_3_O_4_ BioBots were placed in the solution and allowed to stand between 30 and 90 min until the motion started. After this time, particles started to move due to high CO_2_ evolution, which allows an oscillatory buoyancy shift mechanism. Trajectories and propulsion were recorded using a normal smart phone camera and analyses by NIS-Elements software. More details about the propulsion mechanism can be found in the main text and the Supporting Information.

Beer fermentation was carried out by adding 0.5 g of yeast/L as ALG@yeast-Fe_3_O_4_ BioBots or dry yeast to 11°Brix (sucrose grams/100 mL of solution) wort solution measured by an AR4 A KRÜSS analog refractometer. Fermentation was carried out in a closed atmosphere with a septum cap, equipped with a syringe used for gas release and sample acquisition to monitor the fermentation process. The fermentation process was monitored by °Brix determination on 1 mL samples using an analog refractometer. CO_2_ evolution was monitored during fermentation experiments by collecting gas generated in the fermentation flask in a 100 to 250 mL inverted graduated tube submerged in a water bath. The connection between the fermentation flask and the graduated tube was done with a plastic tube. BioBot reutilization experiments were carried out after BioBots were cleaned with water five times to eliminate the excess of yeast generated during the fermentation process. All experiments were carried out in triplicate.

### Statistical Analysis

Brix final values were compared by ANOVA. Mean Brix values were compared at a 0.05 significance level (95% of confidence) using Microsoft Excel software.

### Magnetic Manipulation

BioBot magnetic motion was achieved using a rotating magnetic field of 40 mT. The magnetic setup is formed by a permanent magnet of 40 mT magnetic field attached to a metallic support whose rotation is controlled by an electrical motor. ALG@yeast-Fe_3_O_4_ BioBots were placed in different millimeter channel designs to demonstrate their manipulation and magnetic retrieval form the beer sample. Trajectories and propulsion were recorded using a normal smart phone camera and analyses by NIS-Elements software.

## Supporting Information Available

BioBot catalytic propulsion mechanism details, schematic of porosity modification of magneto/catalytic Janus ALG@yeast-Fe_3_O_4_ BioBots by electrogenerated asymmetric pH gradients, SEM, BET, size distribution, and EDS characterization of ALG@yeast-Fe_3_O_4_ BioBots, Janus porosity effect on the catalytic propulsion, CO_2_ evolution monitoring during the fermentation process, effect of the magnetic field rotating frequency on BioBot magnetic actuation, and reuse of the Janus BioBots in the fermentation process (PDF)
Video S1. Catalytic ALG@yeast-Fe_3_O_4_ BioBot buoyancy shift propulsion (AVI)
Video S2. Janus porosity effect on ALG@yeast-Fe_3_O_4_ BioBot catalytic self-propulsion (AVI)
Video S3. Effect of sugar concentration on Janus BioBot catalytic self-propulsion (MP4)
Video S4. Beer fermentation using Janus ALG@yeast-Fe_3_O_4_ BioBots (AVI)
Video S5. ALG@yeast-Fe_3_O_4_ BioBot magnetic actuation at different frequencies of a rotational 40 mT magnetic field (AVI)
Video S6. ALG@yeast-Fe_3_O_4_ BioBot magnetic manipulation in a complex channel by switching the rotation direction (AVI)
Video S7. ALG@yeast-Fe_3_O_4_ BioBot magnetic retrieval from beer after the fermentation process (AVI)

## Supplementary Material

nn2c12677_si_001.pdf

nn2c12677_si_002.avi

nn2c12677_si_003.avi

nn2c12677_si_004.mp4

nn2c12677_si_005.avi

nn2c12677_si_006.avi

nn2c12677_si_007.avi

nn2c12677_si_008.avi
