# Supplementary material for: Nanostructured Hybrid BioBots for Beer Brewing
Source: ACS Nano. 2023 Apr 12;17(8):7595–603. doi: 10.1021/acsnano.2c12677 (PMC10134490; doi:10.1021/acsnano.2c12677)
Supplement: Supplementary file 1 — nn2c12677_si_001.pdf [file nn2c12677_si_001.pdf]

Supporting Information

Nanostructured Hybrid BioBots for Beer Brewing

*Roberto Maria-Hormigos,[1] Carmen C. Mayorga-Martinez,[2] Tomáš Kinčl,[3] Martin Pumera[1,2,4,5] **

[1]Future Energy and Innovation Laboratory, Central European Institute of Technology, Brno University of Technology (CEITEC-BUT), Purkyňova 123, Brno, 612 00 Czech Republic

[2]Center for Advanced Functional Nanorobots, Department of Inorganic Chemistry, University of Chemistry and Technology Prague, Technická 5, Prague 6, 166 28 Czech Republic

[3]Department of Biotechnology, University of Chemistry and Technology Prague, Technická 5, Prague 6, 166 28 Czech Republic

[4]Faculty of Electrical Engineering and Computer Science, VSB - Technical University of Ostrava, 17. listopadu 2172/15, 708 00 Ostrava, Czech Republic

[5]Department of Medical Research, China Medical University Hospital, China Medical University, No. 91 Hsueh-Shih Road, 40402 Taichung, Taiwan

* e-mail: pumera.research@gmail.com

Catalytic propulsion mechanism details

The forces involved in the catalytic buoyancy shift propulsion reported by Inagi's group are represented in **Equations** (**1–5**):[1]

$$\text{Descending motion: } F = F_{Gravity} - F_{Buoyancy} - F_{Drag} \quad (1)$$

$$\text{Ascending motion: } F = F_{Buoyancy} - F_{Gravity} - F_{Drag} \quad (2)$$

$$F_{Gravity}\ (F_G) = \rho_{particle} * V_{particle} * g \quad (3)$$

$$F_{Buoyancy}\ (F_B) = F_{bubble\ buoyancy} + F_{particle\ buoyancy} \quad (4)$$

$$F_{Drag} = 6\ \pi\ \eta\ \gamma_{particle}\ \upsilon \quad (5)$$

Where $\rho_{particle}$ is the particle density, $V_{particle}$ is the particle volume, g is the constant gravity acceleration, $F_{bubble\ buoyancy}$ is the bubble's contribution to buoyancy force, $F_{particle\ buoyancy}$ is the particle's constant contribution to buoyancy force, η is the medium viscosity, $\gamma_{particle}$ is the particle

radius, and υ the particle speed. So, the changes on the bubble buoyancy forces (as consequence of bubbles trapping and release from the BioBots) is the driven force of the catalytic vertical motion.

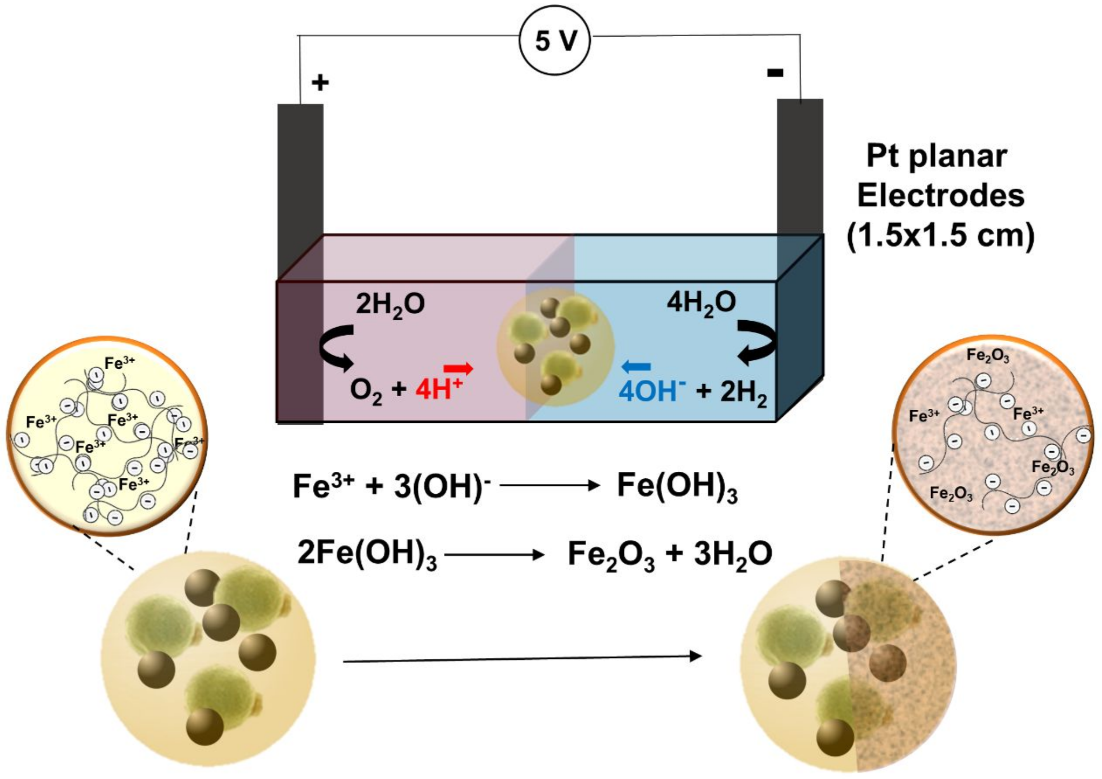

Figure S1. Schematic porosity modification to obtain magneto/catalytic Janus ALG@yeast-Fe_3O_4 BioBots using electrogenerated asymmetric pH gradients.

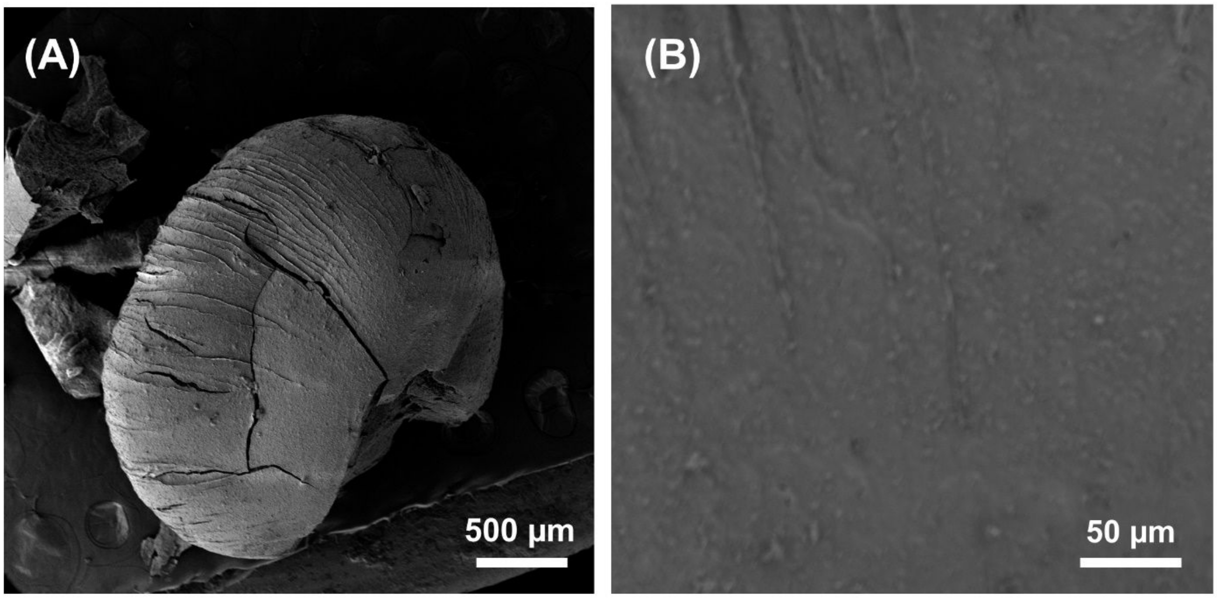

Figure S2. SEM characterization of freeze-dried ALG@yeast-Fe_3O_4 BioBots before porosity modification by the pH treatment. **(A)** Whole particle. **(B)** Surface magnification.

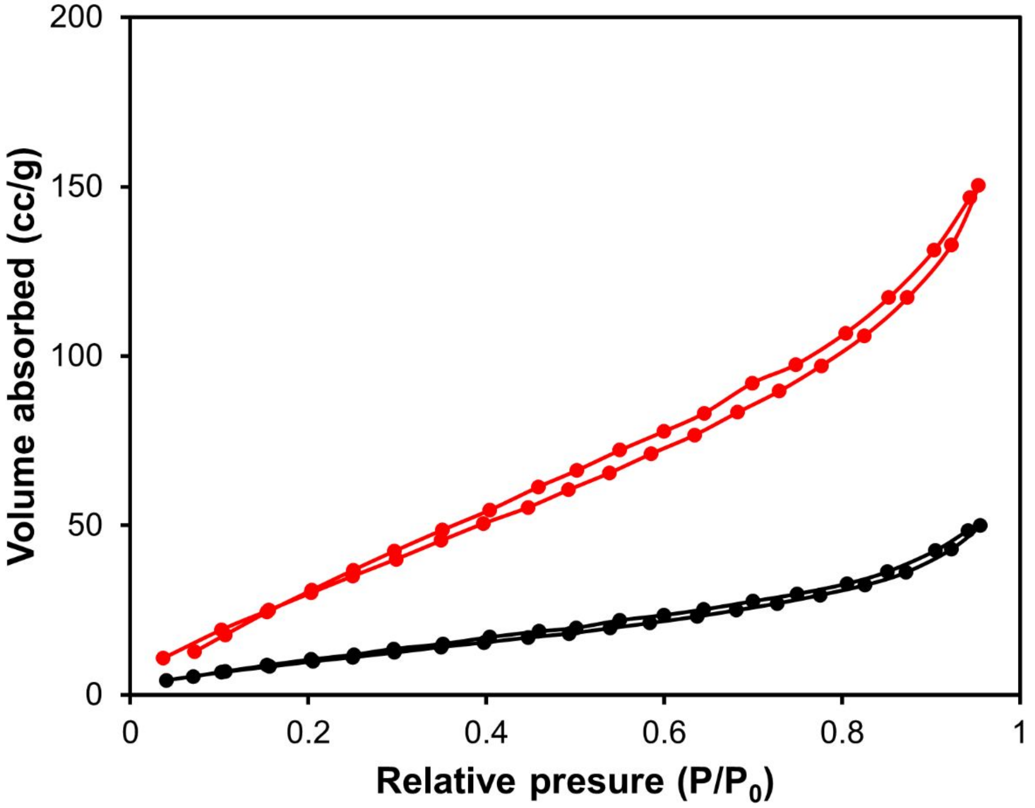

Figure S3. BET characterization of ALG@yeast-Fe_3O_4 BioBots before (black) and after (red) porosity modification by the pH treatment.

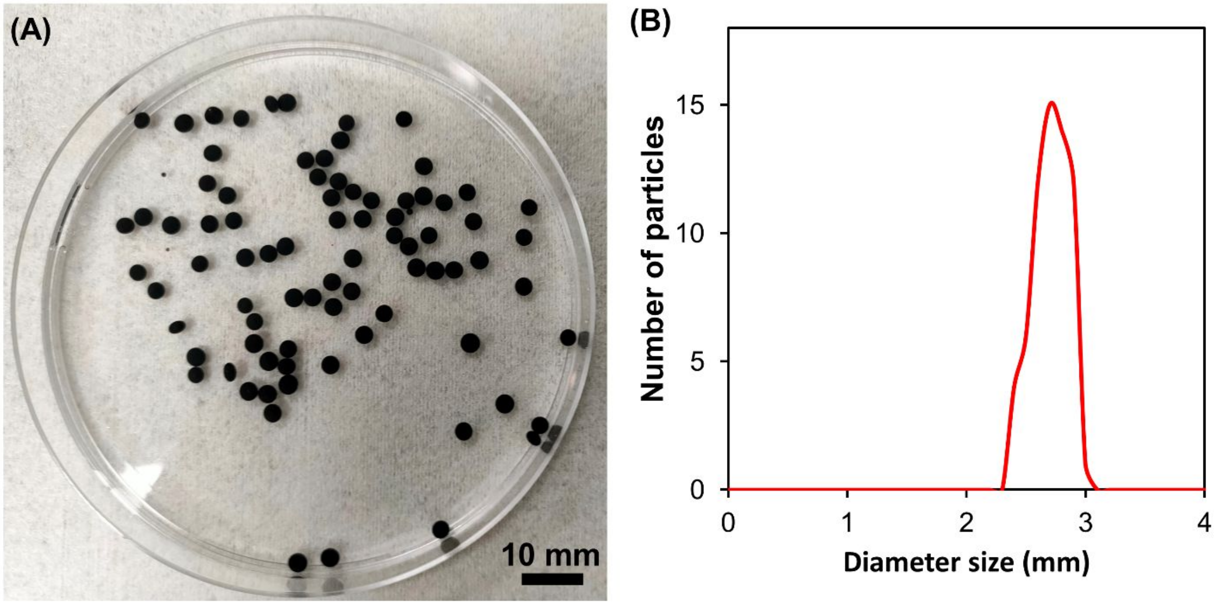

Figure S4. **(A)** Janus ALG@yeast-Fe_3O_4 biobots image and **(B)** corresponding diameter size distribution analysis. Size analysis was performed by NIS-Elements software analysis of the picture after image internal calibration assuming BioBots spherical geometry.

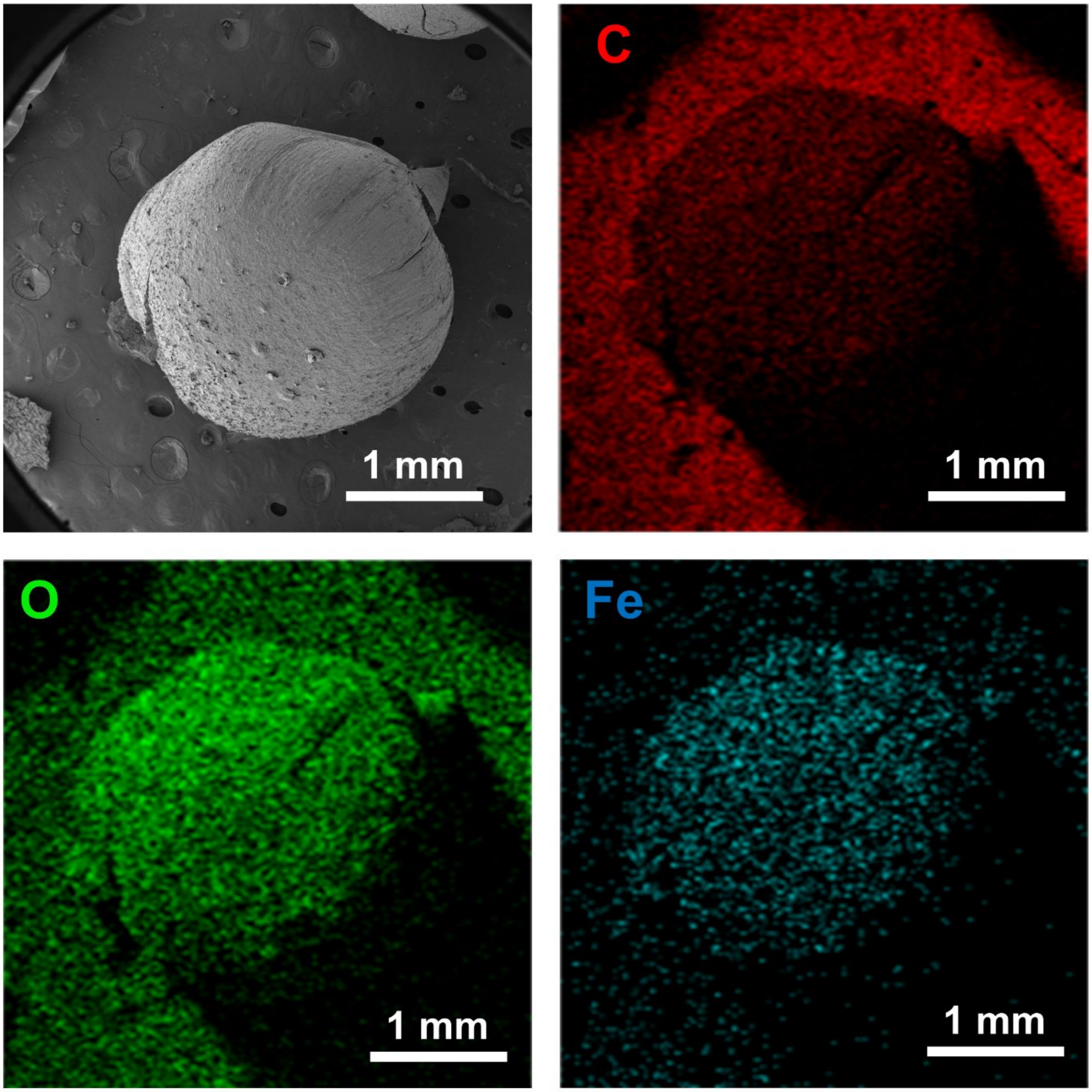

Figure S5. Janus ALG@yeast-Fe_3O_4 biobots SEM image (over carbon tape) and corresponding elemental mapping images from EDS analysis.

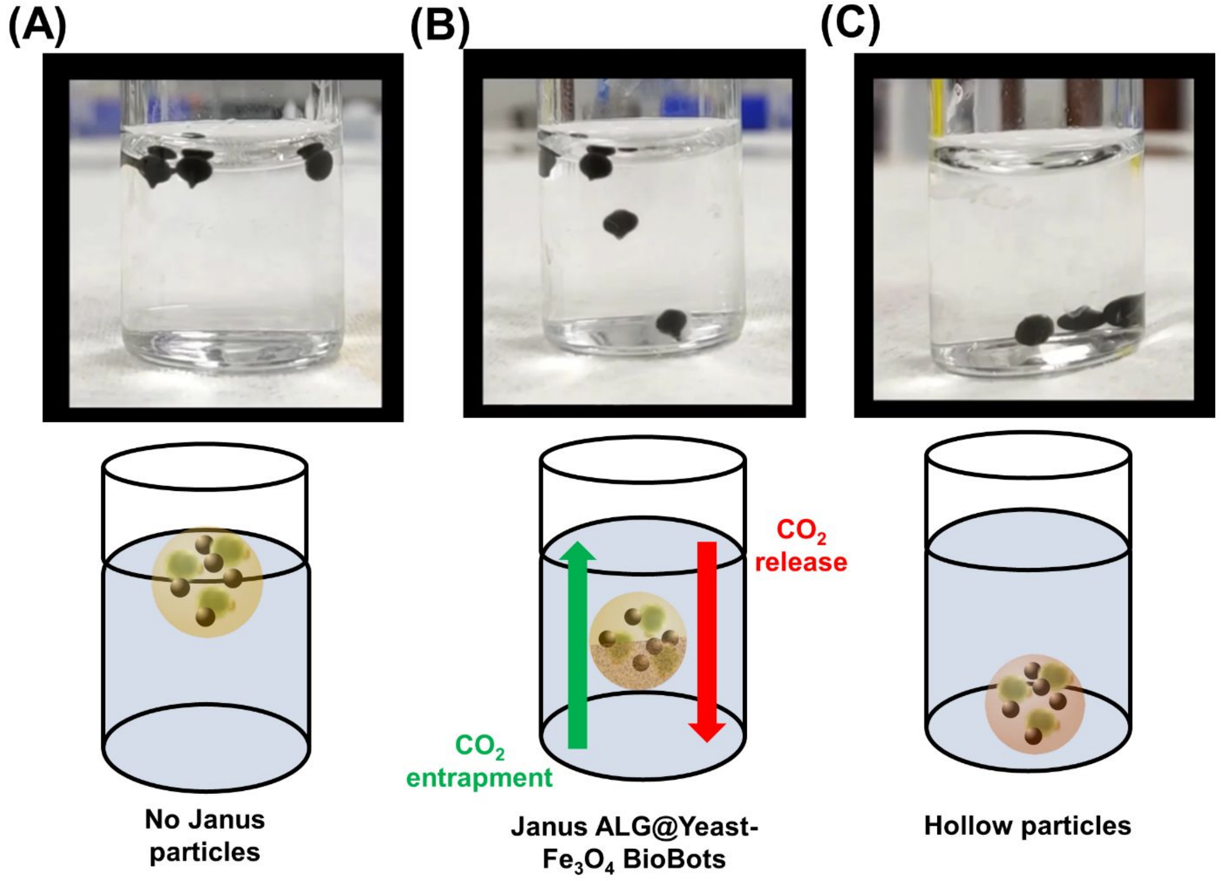

Figure S6. Janus porosity effect on ALG@yeast-Fe_3O_4 BioBots catalytic propulsion. **(A)** Image of non-Janus ALG@yeast-Fe_3O_4 particle before pH treatment. **(B)** Image of Janus ALG@yeast-Fe_3O_4 BioBots after 10 min of pH treatment. **(C)** Image of hollow ALG@yeast-Fe_3O_4 particle after 30 min of pH treatment. Images taken from **Video S2** at a 10 % sugar concentration.

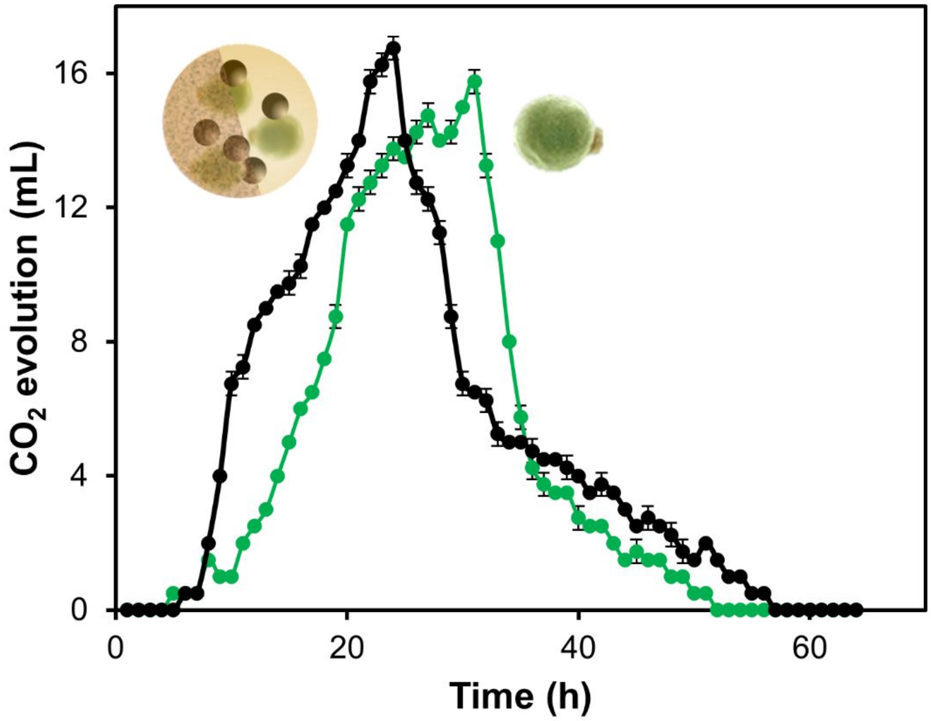

Figure S7. CO_2 evolution monitoring during the fermentation process using Janus ALG@yeast-Fe_3O_4 BioBots (black line) and free yeast (green line). 100 mL solution of wort 11 ˚Brix, inoculation concentration was 0.5 g/L of dry yeast or BioBots, $n = 3$.

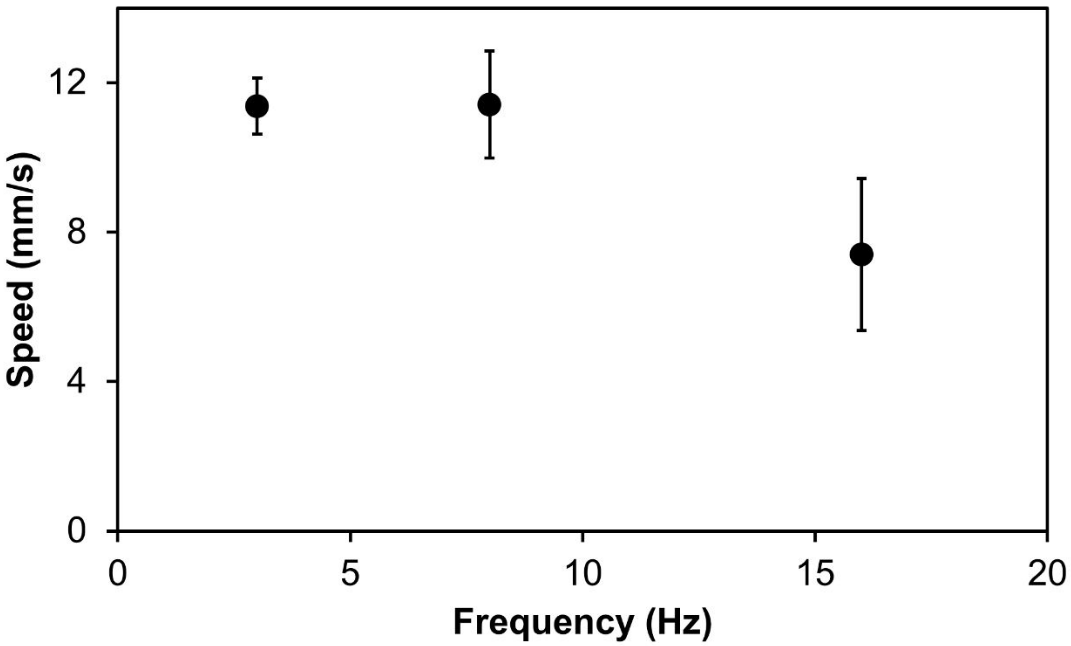

Figure S8. Janus ALG@yeast-Fe_3O_4 BioBots magnetic actuation mean speed and standard deviation of n = 4 at different rotational magnetic field frequencies.

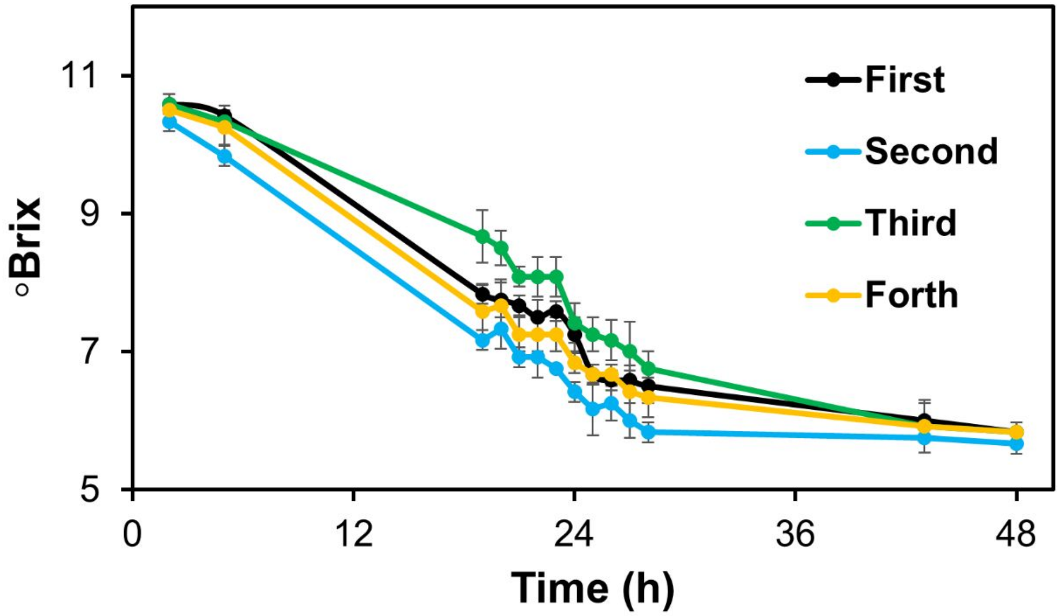

Figure S9. Reuse of Janus ALG@yeast-Fe_3O_4 BioBots. ˚Brix during the fermentation process using the same Janus ALG@yeast-Fe_3O_4 BioBots in consecutive cycles. BioBots were cleaned 5 times with water between cycles to eliminate the excess of yeast produced. Initial wort 11 ˚Brix, inoculation concentration was 0.5 g/L of dry yeast or BioBots, experiments carried out by triplicate, $n = 3$.

REFERENCES

1. Wu, M.; Koizumi, Y.; Nishiyama, H.; Tomita, I.; Inagi, S. Buoyant Force-Induced Continuous Floating and Sinking of Janus Micromotors. *RSC Adv.* **2018**, *8*, 33331.
